# With Our Editors

**Published:** 1906-08-15

**Authors:** 


### WITH OUR EDITORS.

**THE WHEAT AND THE CHAFF.** *"Any position in life, however great or small as the world goes, can be made creditable by its occupant measuring up to the full standard of duty well performed."—Judge J. P. Phillips.*

The above advice is alike applicable to the dental practitioner, to the dental student and to the dental teacher. It matters not whether the reader is a teacher, a student or a practitioner, it is alike true that he who measures up "*to the full standard*" does all that the times and the occasion demand. In order, however, that such standards can be reached, it is also true that each teacher, each student and each practitioner shall sift the "wheat from the chaff," and with a rare pertinacity, smother prejudice, which may perchance be, and often is, individual, and not up to the standard of the best duty which should be performed. In dental progress, it seems to the writer, that such a course is often lost sight of. The status of the profession, the theoretic in the profession, the etiologic in the profession, the improvements in practical dentistry, and the great changes which are constantly taking place in all methods conducive to the cure or amelioration of dental discomfort, are too often considered as vagaries, because of the dislike for careful investigation and because preconceived ideas and methods are so well established, or because a man's reason has been smothered by a preponderance of high-sounding words and names. To all of these we would say that if you measure "*up to the full standard*," that standard must be one acceptable to the intelligent majority of the profession of which you are a member, and if you do not do this, your duty will not be *well performed*.

There never was a time in the progress of the world where individual opinion, if backed by intelligent and educated logic, receives more consideration. I care not what your arguments may be, if its postulates are undeniable, they will receive credence; not all the wisdom of the world is found outside of the dental profession, and new propositions will obtain in our profession credence according to their deserts, and your duty *well performed* will embrace their consideration.

Separate the "wheat from the chaff." Be not the slaves of text-books or dental writers, nor be their unreasoning critics. If writers insist upon the acceptance of hypothesis that are prejudged, born of vague longing, and not the outgrowth of up-to-date economy of science and life, weigh them in the balance, and if found wanting, sweep them along with the chaff. The teacher's duty, the preacher's duty, the practitioner's duty, is not well performed unless it is accomplished without false science or the false ethics of medieval times. The dental reader or teacher is to-day confronted with a lot of chaff among the wheat; *e. g.*, a prominent writer in a prominent journal, *The International,* writes as follows when referring to the education of the dentist: "His training must be given under the guidance of scientific teachers, non-practitioners, men of productive scholarship, men who devote their lives to the special work of teaching the various subjects which are embodied in a medical education, instead of those who give instruction during the intervals of a busy practice." "Without the requisite medical education, the ordinary mechanical expert is not competent to practice his profession." In the foregoing extracts from the pen of Dr. H. P. Carlton, of San Francisco, we have the gist of the argument a number of dental writers, most of whom are members of the Section on Stomatology of the American Medical Association. In the position as stated by Dr. Carlton, surely none can take exception to the statement that we should have "scientific teachers," "men of scholarship" and thoroughly competent for their special work; but when we are informed that they *must be non-practitioners, instead of those who give instruction in the intervals of a busy practice,* we come to the "chaff" of the statement. By whom has it been proven that the practitioner who teaches is not usually the scientific man and the scholar? The intimation is an unjust one, and we also earnestly incline to the opinion, and the opinion is re-inforced by observation and experience, that the dental teachers who are not in active practice soon become imbued with just such "chaff"—become attached to obsolete methods, and are prone to grade themselves a notch higher because relieved of the tedium of actual practice, when in fact they are likely to be retrograding in their ability to teach when compared to the man in active practice. The coterie of "stomatologists" who look askance at the practitioner of dentistry without the M. D. degree, and who believe that if all dentists were first M.D's, the fact *alone* would give us status not now ours, are of the class with Dr. Carlton, and are given also to damn mechanics "with faint praise." We believe that these efforts will make less possible the best returns to our patients whom we serve; and that the "true duty well performed" will be in accepting the triune of theory, science and practice as of equal dignity; and that the fine arts and mechanics enter into all science, into biology, into chemistry, into surgery, into therapeutics, into repair of tissue, just as they originally have done in the creation of tissues. "Separate the wheat from the chaff."—J. D. Patterson, in *Western Journal*.

**RESPECT FOR THE ESSAYIST.** Very frequently we hear complaints from authors of essays presented at state or local meetings that their communications are not sufficiently appreciated by the audiences. This general lack of interest is quite common, and usually manifests itself in non-participation in the discussion following the reading of the essay. It is certainly unjust and very discouraging to the author to meet with such reception. And usually it is the man who has spent much time and energy and ferquently has gone to considerable expense in preparing an original communication which possesses decided merit who meets with such fate. What are the causes of this apparent lack of appreciation? Various sources are responsible for the disappointment. More or less, every one connected with the reading of an essay—viz, the committee in charge, the author and the audience—is at fault. The non-fulfillment of certain well-established principles is the primary cause. Most societies have incorporated in their by-laws a clause reading somewhat like this: The society requires three copies of each paper to be in the hands of the secretary two weeks prior to date of reading. Does the secretary receive these copies? And if not, does he call for them? We have no statistics at our command to prove by figures in what percentage of cases this by-law is complied with, but from actual experience we know that it is a dead letter in most societies. Furthermore, the selection of men, to discuss the paper is often done hap-hazardly, viz., no discrimination is shown in selecting these men according to their qualifications. If the committee in charge would exercise good judgment these troubles are easily avoidable. If, however, the men are selected with their consent and have received a copy of the essay in due time, but are not present at the reading of the paper without a just excuse, they should be severely censured.

The selection of a subject for an essay deserves the closest consideration by its author. It should be borne in mind that practical subjects are always prone to evoke lively discussion, while a purely theoretical essay may fall flat. And still, a theoretical problem will often arouse the interest of the audience provided the author has made a successful attempt to prove his viewpoint by logical reasoning. To rehash a certain theory by copying from a half dozen text-books and presenting this conglomorate mixture in a paper before a society is an insult to the mental status of its members. Recently the writer had occasion to listen to an essay of this nature. The subject of the paper was, "How Do Teeth Erupt?" After recapitulating the various theories *verbatim* from a number of text-books, some of them rather ancient, the writer wound up by questioning the audience, "Did you ever stop to think what makes the hair grow? Did you ever consider why you wink your eye?" Needless to say, the much-bored audience remained silent.

Professional men who attend a meeting owe it to their confrere who presents the essay to be prepared to take part in the discussion. A little thinking about the subject to be discussed a few days prior to the meeting, and consulting the literature on the subject, is always of advantage. It is well to confine one's remarks closely to the point in view, and one should never talk merely to be heard. Just criticism is deserving of the highest praise; it helps to recognize one's own shortcomings. The thorough discussion of an essay is highly satisfactory to every one concerned; the author is pleased because his labor is appreciated, and the audience is thankful for the professional benefit derived.—H. Prinz, in July *Dental Era*.

**BETTER MANAGEMENT OF LARGE DENTAL CONVENTIONS.**

The recent meeting of the Illinois State Dental Society, held in Springfield, has prompted us to a brief consideration of some phases of society meetings in general not with a desire to find fault nor to criticise, but with a feeling of fairness and of mutual helpfulness, that improvement and advancement may be made, not alone in the Illinois Society, but in all societies the country over.

To be of the greatest assistance to the largest number is the true function, and should be the aim of every good dental society. The larger the society becomes, the more difficult becomes the performance of that function. The Illinois Society, after two years of untiring effort on the part of its Committee on Reorganization, finds its membership suddenly trebled. New conditions are at hand, additional tasks for the president, who finds the increased attendance composed of men new in society life, men not trained in society work and proceedings, who know nothing of parliamentary laws and practices. The change that has come about in the society emphasizes the fact that a presiding officer must be, first of all, a parliamentarian, To successfully meet this new condition he must possess the personality the voice, the self-reliance that will command respect, preserve order and so conduct the sessions that the most may be accomplished in the time alloted.

It has been the practice of dental societies, in the past, to choose very frequently a man to fill the president's chair who was utterly lacking in the qualifications of a competent presiding officer, and as a result the societies have suffered. It is a fact that very few men in the societies do, when elected to the presidency, prove good presiding officers, and yet men must be honored with the highest office of the society in recognition of their services to the society, be they parliamentarians are not. What is to be done? Is the whole society to suffer that one man, who cannot ably preside, may be honored? The British Dental Association recently solved this problem. The Association honors a member with the presidency each year, as before, but his duties are limited to the calling to order of the meeting and to the preparation and reading of the presiden'ts address. The gavel is then turned over to a chairman selected by the executive council, because of his qualifications to preside and conduct the meeting successfully. This change has worked admirably with much gain to the British Association and we predict benefit would result if societies generally would adopt this course.

The plan, often adopted, of having those chosen to discuss the papers, write their discussions, we believe not a good one, for such discussions are usually too long, and many times anything but discussions of the paper under consideration. A man when allowed to write a discussion is apt to go astray; the real subject is lost to sight and new ones introduced, all of which results in a loss of time and a subsequent curtailment of the general discussion. We believe that more good comes, especially in a large society, from a free and unlimited extemporaneous discussion of a *few good papers*, than from the necessarily limited discussion of a larger number. As a rule, there are on the programs of the state meetings too many papers for the time allowed for their consideration.

In conclusion, we will mention another mistake too often made, that of allowing a man to appear on the program advocating something he or his friend has to sell. Such a thing is a disgrace to a society and should never be tolerated. By the majority of the men in the profession such a thing is considered an insult and an imposition, and such it is. Suppose supply companies generally could arrange to have their particular products flaunted before societies by arranging with different members to read papers on their different devices and materials. How long would it be before the Dental Society meeting would be a commercial and manufacturing exhibit, pure and simple? There should be no place on the program, no time for such exhibitions of unethical and unprofessional conduct, such brazen effrontery! If a presiding officer has the requisite backbone it is clearly within his province to declare such an attempt out of order and out of place.—Dr. Crouse, in May *Digest.*

